# Marked reduction in hallucination rates with GPT-5: A positive development for medical and scientific writing

**DOI:** 10.51866/lte.1004

**Published:** 2025-11-06

**Authors:** Serkan Polat, Bülent Alyanak, Burak Tayyip Dede, Mustafa Hüseyin Temel, Mustafa Turgut Yildizgören, Fatih Bağcier

**Affiliations:** 1 MD, PM&R, Sivas State Hospital, Sivas, Turkey. Email: dr.serkan.0505@gmail.com; 2 MD, PM&R, Gölcük Necati Çelik State Hospital, Kocaeli, Turkey.; 3 MD, PM&R, Prof Dr Cemil Taşcıoğlu City Hospital, Istanbul, Turkey.; 4 MD, PM&R, Prof Dr Cemil Taşcıoğlu City Hospital, Istanbul, Turkey.; 5 MD, PM&R, Necmettin Erbakan University, Konya, Turkey.; 6 MD, PM&R, Başakşehir Çam Sakura City Hospital, Istanbul, Turkey.

**Keywords:** Large language models, Artificial intelligence, Hallucinations, Medical writing, Publishing

**Dear editor,**

We read with great interest the letter by Jamaluddin et al., which highlighted hallucination as a key challenge in artificial intelligence (Al)-generated writing, particularly in academic and clinical contexts.^1^ Since the publication of their correspondence, developments in large language models (LLMs) have progressed rapidly, with newer systems demonstrating measurable improvements in accuracy. In light of recent performance data for GPT-5, it is timely to revisit this discussion - not to reiterate the risks alone but to acknowledge the notable reduction in hallucination rates and to consider its positive implications for medical and scientific writing.

The launch of GPT-5 has been accompanied by encouraging evidence that one of the most persistent limitations of LLMs - the phenomenon of ‘hallucination’ - can be meaningfully reduced.^2^ Hallucinations, defined as outputs that are factually inaccurate or not grounded in real-world data, have long posed challenges in medical writing, research and clinical documentation.^3^ As highlighted in previous work, such errors may arise from limitations in data training, overfitting or gaps in conceptual understanding, and they become especially concerning when fabricated references or distorted interpretations appear in academic or clinical contexts.^4^

According to recently reported figures, GPT-5 demonstrates a substantial improvement over its predecessors when evaluated with web access. The hallucination rate has decreased to 9.6%, down from 12.9% for GPT-4o - representing a 26% relative reduction. The enhanced reasoning variant, GPT-5-thinking, achieved a rate as low as 4.5%. In addition, GPT-5 produced 44% fewer responses containing at least one major factual error compared with GPT-4o. These advances suggest that improvements in training, reasoning capacity and real-time access to online sources can directly translate into more reliable AI-assisted content generation.^2^

Nevertheless, performance remains context-dependent. When evaluated without internet connectivity on fact-seeking tasks, GPT-5’s hallucination rate increased to 47%, underscoring that human oversight remains indispensable, particularly in high-stakes domains such as medicine. As emphasised by Jamaluddin et al., authors must critically verify Al-generated content and references, while editors and reviewers should adopt proactive screening methods - both manual and software-assisted - to safeguard the accuracy and credibility of publications.^5^

From a broader perspective, GPT-5’s performance trajectory offers optimism for the future integration of LLMs into scientific and clinical workflows. The data indicate that hallucination management is not only possible but also measurable, and sustained progress could make AI tools more dependable partners in knowledge creation. However, realising this potential will require continued collaboration between developers, clinicians and the academic community, with an unwavering commitment to verification and quality control.^4^

In conclusion, GPT-5’s marked reduction in hallucinations represents a meaningful step forward. While challenges remain, the combination of technical advancements and responsible human oversight could transform AI from a promising assistant into a trusted contributor to medical and scientific literature.
